# Hacking cell differentiation: transcriptional rerouting in reprogramming, lineage infidelity and metaplasia

**DOI:** 10.1002/emmm.201302834

**Published:** 2013-07-04

**Authors:** Gonçalo Regalo, Achim Leutz

**Affiliations:** 1 Max-Delbrueck-Center for Molecular MedicineBerlin, Germany; 2 Humboldt-University of Berlin, Institute of BiologyBerlin, Germany; 3 Berlin-Brandenburg Center for Regenerative TherapiesBerlin, Germany

**Keywords:** carcinogenesis, haematopoiesis, metaplasia, transcription factor, trans-differentiation

## Abstract

Initiating neoplastic cell transformation events are of paramount importance for the comprehension of regeneration and vanguard oncogenic processes but are difficult to characterize and frequently clinically overlooked. In epithelia, pre-neoplastic transformation stages are often distinguished by the appearance of phenotypic features of another differentiated tissue, termed metaplasia. In haemato/lymphopoietic malignancies, cell lineage ambiguity is increasingly recorded. Both, metaplasia and biphenotypic leukaemia/lymphoma represent examples of dysregulated cell differentiation that reflect a history of trans-differentiation and/or epigenetic reprogramming. Here we compare the similarity between molecular events of experimental cell trans-differentiation as an emerging therapeutic concept, with lineage confusion, as in metaplasia and dysplasia forecasting tumour development.

## Introduction

Observations of trans-differentiation in tissue culture were initially regarded merely as the result of artificial manipulation, with no physiological correspondence. Meanwhile, reprogramming cell differentiation has advanced as a promising option for future therapeutic approaches. Reprogramming experiments have shown that a few transcription factors may instruct alternative cell differentiation outcomes, yet many of the same factors are often activated in neoplastic tissue that may give rise to cancer. How incoherent cell differentiation states emerge in pre-neoplastic tissue and how they contribute to tumourigenesis is still far from being understood. Clinical observations of epithelial metaplasia and haematopoietic ‘lineage infidelity’ and ‘lineage promiscuity’ are renowned phenomena and biphenotypic leukaemia/lymphoma echo the remarkable data set collected in the past years on experimental cell reprogramming. Here, we review the possibility that in neoplastic transformation aberrantly regulated transcription factors mediate de-/and trans-differentiation steps that may confer selective advantages to the evolving cancer cell. The possibility is discussed that multi-lineage phenotypes and epithelial metaplasia are disease-relevant physiological counterparts of experimental reprogramming.

## Cell lineage definition and reprogramming

**Figure 1:**
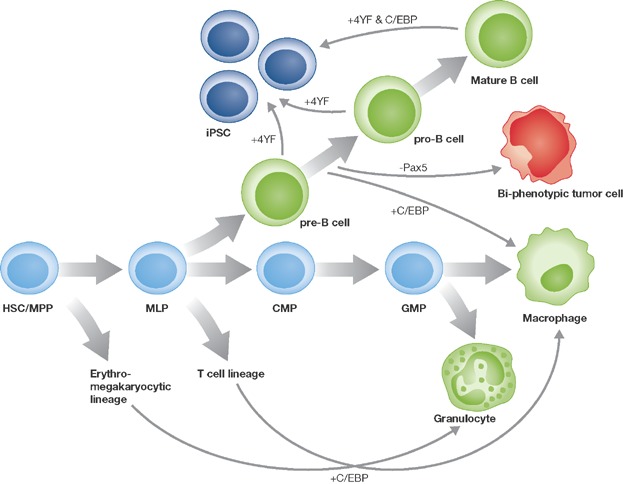
**Schematic presentation of haematopoiesis and experimental trans-differentiation**Simplified partial scheme of haematopoiesis, with emphasis on myelo-B lymphoid lineages. Semi-transparent wide arrows indicate the haematopoietic hierarchy of cell differentiation. Haematopoietic stem cells and multipotent progenitors (HSC/MPP) give rise to cells of the erythroid/megakaryocytic lineage and to myelo-lymphoid precursor cells (MLP). The adaptive immune B-cell and T-cell lineages emerge and common myeloid progenitors (CMP) give rise to cells of the innate immune system through granulocyte/macrophage progenitors (GMP) that may differentiate into the respective functional end cells (various types of granulocytes, monocytes and dendritic cells; not shown for simplification). C/EBPs may trans-differentiate erythro/megakaryocytic precursors, T cells and early B cells into inflammatory macrophages (grey curved arrows). Loss of Pax5 may generate various types of myeloid cells and loss/reduction of Pax5 in B cells may promote neoplastic transformation. Early B cells may be reprogrammed into induced pluripotent stem cells (iPS) by the four ‘Yamanaka transcription factors’ (4YF: Oct4, Sox2, Klf4, c-Myc), whereas late B cells require additional C/EBP for iPS reprogramming.

Previously scorned as tissue culture artefact, the concept of cellular reprogramming meanwhile struggled its way to mainstream scientific acceptance. The field of cell reprogramming entails the potential to revolutionize pharmacological screening approaches and feeds hopes for development of new cell based therapies for degenerative diseases. In a breakthrough experiment, genes coding for four transcription factors (Oct4, Sox2, c-Myc and Klf4) were ectopically expressed in normal fibroblasts, granting them properties very similar to embryonic stem cells (Takahashi & Yamanaka, 2006). Under appropriate cell culture conditions, iPS cells can differentiate into cells that resemble functional, mature cell types of various lineages. Before the landmark study of the Yamanaka lab, pioneering work of the late eighties and early nineties of the last century already revealed that the expression of one or few transcription factors were sufficient to alter the differentiation fate of a cell. Most strikingly, the expression of MyoD was shown to induce skeletal muscle differentiation in several cell types (Weintraub et al, 1989). Most of the previous knowledge on reprogramming, however, came from the haematopoietic system. During the course of haematopoiesis, cell differentiation appears to follow well-defined and controlled paths from haematopoietic stem cells (HSC), to early progenitors with restricted self-renewal capacity, to precursor cells of one of the at least eight haematopoietic cell lineages (Fig 1). Each differentiation stage is characterized by distinct transcription factor signatures that function in networks to define cell lineages (Orkin & Zon, 2008). Transcription factors, including Runx1, C/EBPα, C/EBPβ, GATA1, Myb, E2A, PAX5, Tal1/SCL or PU.1, are expressed or shut-off in an orchestrated manner to achieve blood cell lineage specification. Failure to tightly regulate those factors may ultimately result in the development of leukaemia or lymphoma. Key lineage transcription factors work in combination with co-factor complexes both through the activation or silencing of lineage-specific genes and concomitantly by blocking the activity of competing transcription factors of other lineages. This may be achieved by physical interaction between transcription factors, leading to combinatorial regulation, selective neutralization or reshaping of gene regulatory protein complexes. Examples of transcription factor duplicity are numerous and include GATA1 and PU.1 in erythro/megakaryopoiesis, or C/EBP/PU.1 and C/EBP/Myb in myelopoiesis, respectively, or C/EBP and GATA1 in granulopoiesis (Graf, 2002; Kim & Bresnick, 2007; Ness et al, 1993). Collaborative and competitive functions of several transcription factors also display quantitative traits. High levels of PU.1, for instance, promote macrophage differentiation, while lower levels promote B-cell formation. Interestingly, experimental reduction of PU.1 in myeloid cells incites their leukaemic conversion (Rosenbauer et al, 2004). Reduced expression of Pax5 in early B cells provokes emergence of biphenotypic lympho/myeloid cells, as seen during embryonic development and in acute lymphoblastic biphenotypic leukaemia (Simmons et al, 2012). Similarly, altered translation initiation sparing some of the of GATA1 or C/EBPα N-termini are connected to leukaemogenesis, suggesting balanced and context specific functions in homeostasis and calling into attention the relation between lineage specifying factor dysregulation and disease (DeKoter & Singh, 2000; Ge et al, 2006; Pabst et al, 2001).

With the emerging concept of lineage-specifying transcription factors, the question arose of what would be the outcome of inappropriate activation or inactivation of ‘lineage-specific’ transcription factors. The results of many experiments clearly showed that lineage committed precursors were more flexible than initially anticipated, and in several cases gave rise to alternative cell types. Studies with GATA1 for instance, showed that ectopic expression in myeloid progenitors or common lymphoid progenitors redirected their development into the megakaryocytic/erythroid lineages (Iwasaki et al, 2003; Kulessa et al, 1995). PAX5 deficient B cells, when expanded in tissue culture, were found to become ‘undecided’ and adopted phenotypes of various haematopoietic lineages (Nutt & Busslinger, 1999). Interestingly, a similar role of another PAX family member, PAX7, has been suggested in cell fate decision in the pituitary gland. In this model, PAX7 deficient precursors are redirected from one secretory cell type, the melanotropes, into another, the corticotropes. The potential of PAX7 to mediate cell-fate decisions in the pituitary gland is related to its chromatin-remodelling role and indicates that differentiation mechanisms are analogous in different tissues (Budry et al, 2011).

A remarkable reprogramming and trans-differentiation potential was discovered for C/EBP transcription factors (Fig 1). Early studies showed that expression of C/EBPβ together with Myb in fibroblast led to the activation of myeloid genes (Ness et al, 1993) and expression in haematopoietic progenitors caused granulocytic differentiation (Muller et al, 1995). Ectopic expression of C/EBPα or C/EBPβ in B- or T lymphocytic precursors induced their trans-differentiation into macrophages. Reprogramming resulted from repression of Pax5 by C/EBP to silence B-cell identity and collaboration between C/EBP and PU.1 to activate myeloid genes (Feng et al, 2008; Xie et al, 2004). Thus, similar to normal haematopoiesis, lympho-myeloid reprogramming involves activation of lineage specific genes and repression of genes of competing lineages. Interestingly, the C/EBP bZip domain is known to bind to CpG methylated DNA (Rishi et al, 2010) and trans-differentiated myeloid cells maintained the B lineage specific DNA methylation pattern (Rodríguez-Ubreva et al, 2012). These results indicate a dominant role of histone modification and chromatin remodelling during lymphoid–myeloid conversion, although completion of reprogramming was accompanied by changes in the methylation pattern of some genes (Kallin et al, 2012).

Beyond pure academic interest, recent developments have now fuelled hope of direct reprogramming of one cell type into another lineage as an alternative and circumventing the induced pluripotent state. Direct reprogramming might provide the quintessential therapeutic answer to some of the most afflicting illnesses of our time (Robinton & Daley, 2012; Vierbuchen & Wernig, 2012). A notable example is the potential for treatment of diabetes mellitus type 2 by *in vitro* originating insulin producing islet cells. Exocrine pancreatic cells, as well as keratinocytes, fibroblasts and hepatocytes were found to be reprogrammable into insulin producing cells by transcription factors including Pdx1, MafA and Ngn3 (Aviv et al, 2009; Motoyama et al, 2009; Tateishi et al, 2008; Zhou et al, 2008). Insulin producing cells were also reprogrammed from circulating bone marrow cells in a rat model by repressing Sonic Hedgehog and Rest/Nerf, and expressing the ‘pancreatic’ transcription factor Pdx1 (Li et al, 2012). In myocardial infarction, scarred cardiac tissue cannot recover its previous contractile properties due to the limited regenerative potential of cardiac cells. Two research groups have recently found that heart cells can be obtained by ectopic expression of three transcription factors, GATA4, MEF2C and TBX5. The groups of Srivastava and Olson have managed to reprogram even resident, dividing non-cardiomyocytes by retroviral gene transfer into functional myocytes, greatly improving cardiac function upon injury (Qian et al, 2012; Song et al, 2012). In neurological diseases that are connected to the incapacity of neurons to self-renew (reviewed in Rouaux et al, 2012) reprogrammed neurons were obtained by expression of Ascl1, Brn1 and Myt1l (Pang et al, 2011; Vierbuchen et al, 2010) and dopamine-producing neurons were produced by transfecting human and mouse fibroblasts with a set of three transcription factors (MASH1, NURR1 and LMX1a), with remarkable implications for the treatment of Parkinson's disease (Caiazzo et al, 2011). A single transcription factor, Oct4, was shown to generate haematopoietic stem cells from fibroblasts that could be further differentiated into various myeloid cell types, suggesting functionality of all intermediate precursor states and lineage commitment steps (Szabo et al, 2010).

Despite all promises of experimental reprogramming, scanning the literature for physiological naturally occurring equivalents of cell fate plasticity reveals a much darker side of the concept. One where transforming cells in cancer development may profit from lineage confusion by aberrantly expressed transcription factors, promoting tumourigenesis. Examples of this are found in leukaemia and in epithelial trans-differentiation, or metaplasia.

## Haematopoietic lineage infidelity and tumourigenesis

Some 2–5% of aggressive types of leukaemia and lymphoma are characterized by blast populations that simultaneously express myeloid and lymphoid lineage markers (Swerdlow et al, 2008). The clinically biphenotypic entity is not to be mistaken with acute bi-lineal leukaemia, which is assigned to more than one population of blasts of different lineages (Weinberg & Arber, 2010). The inter-lineage heterogeneity of biphenotypic leukaemia may be the result of ‘lineage promiscuity’, meaning that the leukaemia originates from precursor cells that maintain the potential to differentiate into alternative lineages, or from reprogramming events induced by the oncogenic process resulting in ‘lineage infidelity’ (Bagg, 2007; Lee et al, 2008). Translocations involving the chromosomal segment 11q23 involving the mixed-lineage leukaemia gene (MLL) are frequently associated with this pathology. Dozens of MLL translocations have been identified and the ability to induce an ambiguous phenotype seems to depend in part on the MLL-fusion partner, with MLL-ENL and MLL-AF4 being associated with the biphenotypic outcome. Although not yet fully uncovered, the mechanistic explanation for the phenotypic ambiguity of MLL leukaemia might be assigned to the disturbance of a transcriptional progenitor signature (Krivtsov et al, 2006). MLL is homologous to the Drosophila trithorax gene and both protein products play important roles in epigenetics by perpetuating the chromatin structure through propagating pre-set gene expression signatures, thus conveying the epigenetic information to the progeny. MLL is part of large chromatin modifier complexes and entails histone methyltransferase activity that modifies histone H3 lysine 4 (H3K4) and H3 lysine 79 (H3K79) that leads to gene activation and transcription elongation, such as of HOX genes that are crucial for organization of the body axis and cell lineage definition (Southall et al, 2009).

Early demonstration of B-cell to myeloid reprogramming was achieved by retroviral encoded signalling oncogenes, such as Ras or Raf that also activate C/EBPβ (Klinken et al, 1988). Later experiments showed that removal of PAX5 opened way for trans-differentiation into various myeloid cell types and to leukaemia development (Nutt et al, 1999). Indeed, modulation of PAX5 levels in lymphoid progenitors can direct differentiation into fully mature B cells, or in case of low PAX5 expression, to an intermediate biphenotypic state (Fig 1), very similar to the one found in biphenotypic leukaemia (Simmons et al, 2012). As C/EBPβ is expressed at low level in B cells, it is possible that alterations of C/EBPβ functions by upstream signalling events may promote myelo-lymphoid lineage confusion and leukaemic conversion (Hsu et al, 2006). CEBPα gene mutations may not only abolish the proliferation suppressive function of the full-length p42 CEBPα, but also correlate with the expression of lymphoid markers in AML cases (Wouters et al, 2008), suggesting that myelo-lymphoid lineage ambiguity could be achieved in both lineages by converging routes.

Markers of various haematopoietic cell lineages have also been observed in multiple myeloma (Epstein et al, 1990) and a continuity of B-cell to myeloid/osteoclastogenic conversion has been considered (Silvestris et al, 2009). Myeloid differentiation programs also become frequently activated in T-cell based anaplastic large cell lymphoma (ALCL), where C/EBPβ plays an important role in sustaining proliferation (Anastasov et al, 2010; Jundt et al, 2005). Lineage plasticity and trans-differentiation from common acute B lymphoblastic lymphoma to myeloid histiocytic/dendritic and Langerhans' cell sarcoma has been observed during disease progression (Feldman et al, 2008). Moreover, a fraction of the B-cell program is extinguished in Hodgkin's lymphoma cells and transcription factors (including C/EBPβ) required for dendritic cells, natural killer and T cells, and thus contributing to lineage infidelity, are up-regulated (Janz et al, 2006). It is therefore conceivable that deregulated C/EBPβ is involved in mediating the mixed lineage phenotype and that the mixed phenotype is part of the cause, rather than a consequence of malignant progression.

## Trans-differentiation and epithelial metaplasia

For a time, research sought evidence of physiological reprogramming or trans-differentiation, as a proof that it not only represents an experimental artefact, but also occurs in the organism. It was clear from early on, that if somewhere, physiological reprogramming would be encountered in the context of tissue damage and regeneration. The plasticity of pancreatic cells in the regenerating pancreas has been know since the early eighties, in particular their ability to trans-differentiate into hepatocytes *in vivo* (Shen et al, 2003). A recent report shows that hepatocytes undergo widespread Notch-dependent reprogramming into biliary epithelial cells upon injury. In this report it was suggested that reprogramming is a common feature of liver regeneration (Yanger et al, 2013).

**Figure 2:**
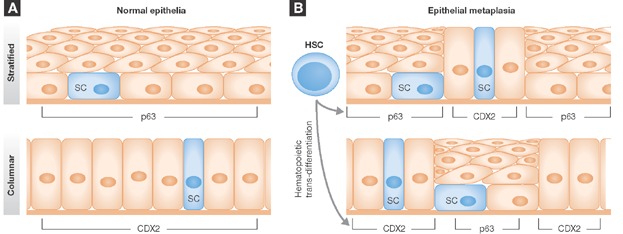
**Schematic presentation of metaplasia and trans-differentiation**
Normal stratified and columnar tissue differentiation requires the expression of p63 and CDX2, respectively.Under stress conditions such as during injury and regeneration, or chronic inflammation, ectopic expression of CDX2 in stratified cells induces columnar/intestinal differentiation amidst stratified epithelium, as it happens in metaplastic Barrett's oesophagus. Conversely, ectopic p63 expression leads to abnormal stratified differentiation amidst normal columnar epithelium, such as it happens in cigarette smoke induced stratified metaplasia of the lung. In both scenarios circulating bone marrow derived stem cells may also provide a source of these metaplastic foci in an effort to replace exhausted committed epithelia tissue-specific stem cells.

Several groups attempted to show that circulating haematopoietic cells were capable of trans-differentiation, but this was subsequently contested mostly as the reflection of extremely rare events and quite often related to cell fusion, instead of true lineage conversion (Wagers & Weissman, 2004). Nevertheless, Houghton and collaborators showed that gastric cancer may originate from imperfect trans-differentiation of bone marrow derived stem cells into epithelial cells (Fig 2) in a mouse model of chronic Helicobacter infection (Houghton & Wang, 2005). Later, this concept was extended to the onset of oesophageal cancer (Hutchinson et al, 2011). This work highlighted that under stress conditions and enhanced tissue regeneration, circulating haematopoietic cells may indeed trans-differentiate as a mechanism that compensates for exhaustion of tissue specific stem cells. Interestingly, the authors also showed that the imperfect trans-differentiation of bone marrow cells into epithelial cells leads to neoplasia. In their mouse model neoplasia is progressive and encompasses an intermediate step of lineage ambiguity that precedes the occurrence of dysplastic and neoplastic lesions, typically accounted as metaplasia.

Known to pathologists for decades, metaplasia is a clinical entity defined as the transformation of patches of a differentiated epithelium into another type of differentiated epithelium (Slack & Tosh, 2001). Associated with chronic exposure of epithelial tissue injury, metaplastic foci are thought to represent sites of cancer initiation. Although poorly studied, the development of metaplastic lesions has been shown to depend on *de novo* expression of key transcription factors that re-direct epithelial differentiation to a different fate, similar to biphenotypic haematopoiesis and to experimental reprogramming. Accordingly, pathologically relevant trans-differentiation and mixed lineage features might not be reserved to leukaemia/lymphoma but might also be relevant to epithelial neoplastic transformation.

Adult epithelial tissues, similar to its haematopoietic counterparts, arise through highly controlled mechanisms of cell differentiation. Somatic stem cells, embedded in the tissues and sheltered in specialized niches of mesenchymal cells, give rise to progeny that will ultimately differentiate into functional cells to repopulate the tissue. The specific outcome of this differentiation process depends on transcription factor networks and on cellular signalling pathways, mostly controlled by neighbouring cells, defined during embryogenesis, and persisting in adulthood as regenerative potential (Blanpain et al, 2007; Orkin & Zon, 2008). Metaplasia appears as a subversion of this system, leading to the emergence of a trespassing tissue amidst another kind of differentiated tissue. An example is intestinal metaplasia of the stomach, consisting of the abnormal development of intestinal epithelium amid normal stomach epithelium. Another example is Barrett's oesophagus, where squamous epithelium is replaced by tissue with intestinal features. Conversely, squamous metaplasia of the lung appears when ciliated respiratory epithelium acquires features of stratified epithelium. Likewise, it has been shown that pancreatic acinar cells can transdifferentiate into beta cells, in a preneoplastic event coined acinar-to-ductal metaplasia (Husain & Thrower, 2009).

If in experimental reprogramming artificial induction of master switch transcription factors and selection of reprogrammed cells is mandatory to the success, how would comparable changes be physiologically attained in trans-differentiating metaplastic epithelium? The consensus is that metaplasia is intimately related to chronic injury, inflammation, regeneration and healing, as hinted by the *in vivo* plasticity of pancreatic and hepatic cells. For instance, squamous metaplasia of the lung is associated with long-term exposure to cigarette smoke, gastric acid reflux with Barrett's oesophagus, *H. pylori* infection with intestinal metaplasia of the stomach, and pancreatitis triggers the development of acinar-to-ductal metaplasia (Husain & Thrower, 2009; Playford, 2006; Randell, 2006; Smith et al, 2006). In line with this, continued use of anti-inflammatory drugs may protect from the development of metaplastic lesions and progression to cancer (Vaughan et al, 2005). As it is also known that inflammation can have profound effects on epithelial gene expression, one could envisage that inflammation alters epigenetic functions to permit master switch transcription factor deregulation that finally leads to reprogramming of the epithelium. Additionally, chronic injury and inflammation have thorough effects on tissue architecture and microenvironment. Metaplastic tissue is usually infiltrated with inflammatory cells, which secrete numerous cytokines and inflammatory factors. Illustrating the relevance of the association between inflammation and reprogramming, overexpression of the cytokine IL1-β in the gastric mucosa is sufficient to induce metaplasia and gastric cancer in 70% (Tu et al, 2008). Cytokines such as IL1-β may therefore play a key role in epithelial reprogramming by either altering the epithelial–mesenchymal crosstalk, or by inducing changes in the expression and/or activity of key transcription factors that drive trans-differentiation.

Metaplasia also generally occurs between cell types that arise from neighbouring regions in the adult or in the developing embryo (Slack, 2009). As in haematopoietic development, related cell types differ by only few master regulator genes, suggesting a higher propensity of re-directing cell fate (Yuasa, 2003). An illustration of this is that abnormal differentiation of gastric stem cells located in the gastro/oesophageal transition has been suggested to give rise to Barrett's oesophagus in an IL1-β-induced mouse model of oesophageal cancer (Quante et al, 2012).

In any case and independently of the onset mechanisms, metaplastic tissue is thought to promote appearance of neoplastic foci, although it remains unclear how emerging tumour cells take advantage of lineage confusion for their own benefit. A recent set of publications has now disclosed that tumours, despite presenting markers from a given tissue, do not forcibly derive from stem cells specific for that given tissue. The group of Blanpain has highlighted the plasticity of epidermal stem cells, by showing that basal cell tumours, despite expressing hair follicle markers in the early stages do not derive from hair follicle progenitors, but from basal stem cells that are reprogrammed into hair follicle progenitors. As basal cell tumourigenesis is WNT dependent, and basal stem cells carry a number of WNT inhibitors, reprogramming might allow the cancer cells to circumvent this inhibitory effect (Youssef et al, 2012). Likewise, despite the basal nature of BRCA1 derived breast tumours, it was shown that they do not derive from basal progenitors, but rather from luminal progenitors that acquire basal-features (Molyneux et al, 2010). To what extent this phenomenon reflects a general event in tumour progression is still unclear. It is nevertheless reminiscent to leukaemic ‘lineage infidelity’ and tempting to speculate that epithelial metaplasia reflects analogous mechanisms. This suggests that genetic and epigenetic changes underlying metaplastic transformation, as well as alterations in mesenchymal/epithelial crosstalk promoted by inflammation, represent pre-cancerous events, which stresses the clinical importance and the urgency to elucidate the molecular mechanisms of metaplasia.

## Examples of deregulated epithelial transcription factors and trans-differentiation

P63: is a transcription factor that shares high similarity with its eminent homolog, the p53 tumour suppressor gene. P63 deficient mice display a striking generalized defect in stratified epithelium, indicating crucial roles in maintaining stem cell populations in squamous and other stratified epithelia (Yang et al, 1999). Later analyses substantiated the role of p63 in squamous metaplasia development. Bronchiolar metaplasia and squamous-cell pulmonary carcinomas show strong p63 nuclear immunostaining (Wang et al, 2002). Other metaplasias involving squamous reprogramming, such as Barrett's oesophagus or cervical squamous metaplasia, also display strong p63 expression (Roman et al, 2007), which may grant p63 features of a squamous ‘master switch’ gene (Fig 2).

Although associated with the expression of squamous keratins and differentiation markers, the mechanism of how p63 drives squamous differentiation remains largely unknown. A recent report suggested that p63 deficient mice develop Barrett's oesophagus not through transdifferentiation events, but by competition between stem sell lineages, and reactivation of residual embryonic stem cells with alternative differentiation potential (Wang et al, 2011). Nonetheless, p63 was shown to repress C/EBPδ in human keratinocytes, and a coherent crosstalk was observed between C/EBPβ and p63 in the regulation of terminal squamous differentiation (Borrelli et al, 2007; Pozzi et al, 2009). Compound deletion of C/EBPα/β was also shown to increase p63 levels, and simultaneously induce keratinocyte hyperproliferation (Lopez et al, 2009). A recent report further substantiated the role of C/EBP transcription factors in the process of squamous metaplasia of the lung, as mice with a conditional deletion of C/EBPα in the lung developed squamous metaplasia in adulthood, together with other features of chronic obstructive pulmonary disease, COPD, although no p63 connection was mentioned (Didon et al, 2010). Nonetheless, this data suggests that C/EBP transcription factors play a major role in normal and metaplastic squamous differentiation downstream of p63.

CDX2: is a homeobox transcription factor that together with its homolog CDX1, plays an important role in the development and specification of gastrointestinal tissues. In adulthood, both CDX1 and CDX2 are predominately expressed in the colon and small intestine and are absent from the stomach and the oesophagus (Barros et al, 2010; Suh & Traber, 1996). CDX2 is strongly expressed during the transformation of endoderm into typical intestinal columnar epithelium (Stringer et al, 2008). Moreover, mice with conditional deletion of CDX2 in the endoderm fail to develop a functional intestinal epithelium and present features of oesophageal-like stratified epithelium (Beck et al, 1999; Fig 2). This critical role of CDX2 in intestinal development has been associated with its ability to regulate a vast array of intestinal genes, including TFF3, MUC2, ALPI or VIL1 (Colleypriest et al, 2010).

The initial connection between CDX2 and metaplasia was established by the observation that, intestinal metaplasia of the stomach and Barrett's oesophagus, both express CDX2 (Bai et al, 2002; Eda et al, 2003). Later studies have lent genetic support to the metaplasia-CDX2 connection. Forced expression of CDX2 under a gastric specific promoter in mice lead to substitution of the gastric mucosa with functional intestinal epithelium, although a similar study failed to fully abet the intestinalization of oesophageal epithelium (Kong et al, 2011; Silberg et al, 2002). CDX2 induces expression of intestinal markers in gastric or oesophageal cell lines upon transfection (Barros et al, 2010; Colleypriest et al, 2010). Several factors also known to cause intestinal metaplasia do so through up-regulation of CDX2 as in the case of BMP4, a member of the TGF-beta super family (Barros et al, 2010). Interestingly, IL1-β and TNF-α activate the CDX2 promoter with concomitant expression of intestinal markers (Kazumori et al, 2006; Wong et al, 2005), which buttresses the connection between metaplasia and inflammation and the notion that master switch gene products, such as CDX2 or C/EBPs, are regulated by cytokines and inflammatory stimuli (Tsukada et al, 2011).

SOX9: is a member of the SOX family of transcription factors, and plays a wide role in embryogenesis and the differentiation of several tissues. SOX9 is ectopically expressed in acinar-to-ductal metaplasia together with HNF6, repressing acinar genes and inducing ductal differentiation in acinar cells (Prevot et al, 2012). Acinar cells from transgenic mice that ectopically express SOX9 display traits of ductal differentiation. Furthermore, upon pancreatic injury these transgenic mice develop acinar-to-ductal metaplasia at higher frequency than control mice, and importantly SOX9 was shown to be mandatory for the progression of metaplastic cells into pancreatic intra-epithelial neoplasia (Kopp et al, 2012).

## Genetic and epigenetic alterations

Although the induction of signalling pathways by chronic inflammation in both epithelial and mesenchymal cells might play a role in the early development of metaplasia, genetic and/or epigenetic alterations are likely required for prolonged maintenance of a reprogrammed state that eventually progresses into tumourigenesis. The effects of inflammation are difficult to analyse and quantify, however, it is evident that chronic inflammation has deleterious effects on stem cell equilibrium (Quante & Wang, 2008). This effect is likely achieved through changes in the cellular microenvironment and stem cell niche composition. For instance, dendritic cells are increased in Barrett's oesophagus in response to inflammation, and it was suggested that this activates ‘dormant stem cells’ through the induction of Musashi1 expression that alter cell differentiation programs, inducing metaplasia (Bobryshev et al, 2010). The accumulation of inflammatory cells and cytokines, together with the accelerated tissue renewal rates may increase the chance of epigenetic variance that eventually cause lineage infidelity. Also, chronic inflammation is thought to contribute to genetic instability and metaplastic conversion, as reflected by the presence of microsatellite and chromosomal instability and loss of heterozygocity (Gleeson et al, 1998; Zaky et al, 2008). In fact, prolonged exposure to *H. pylori* was shown to lead to alteration of DNA-repair machinery and to a ‘mutator’ phenotype. How exactly *H. pylori* infection induces genetic instability, however, remains to be resolved. Yet, large amounts of bio-reactive free-radicals are produced in the context of chronic inflammation that may cause DNA damage (Machado et al, 2010) and extend the association to the mononuclear phagocyte system.

An additional, unexpected connection between host–pathogen interaction and physiological reprogramming thus emerges: intracellular parasites have long been suspected to remodel host cell gene expression in order to achieve an optimal niche to complete parasite life cycle. Masaki and collaborators have now shown that the Leprosy Bacilli reprograms Schwann cells to a state of mesenchymal progenitor-like cells. This mechanism entails epigenetic changes at the DNA level of the reprogrammed cell, and promotes bacterial dissemination (Masaki et al, 2013). In view of these data, and considering that *H. pylori* is often suggested to be a facultative intracellular parasite (Dubois & Boren, 2007), it is tempting to speculate about a mechanism of chronic *H. pylori* infection and gastric metaplastic reprogramming.

Mutations in tumour suppressor genes, including P53 and APC or c-MYC amplification as observed in intestinal metaplasia or Barrett's oesophagus, are considered as consequences of carcinogen/inflammation-induced genetic instability (Dolan et al, 1999; Rygiel et al, 2008; Segal et al, 2004). While recurrent genetic alterations have never been consistently demonstrated in metaplasia, DNA methylation as an epigenetic modification has been suggested to affect the expression of reprogramming genes in metaplastic lesions. Chronic inflammation has been associated with aberrant DNA methylation patterns in several tissues. This may be ascribed to the activity of cytokines and inflammatory by-products that lead to DNA halogenation, a condition that mimics endogenous methylation (Valinluck & Sowers, 2007). Accordingly, chronic exposure to hydrochloric acid and bile acids, important components of gastro-oesophageal reflux, were shown to lead to demethylation of the promoter of the CDX2 gene, increased promoter activity and expression of the protein in Barrett's oesophagus (Liu et al, 2007).

Few reports have specifically addressed the subject of histone modifications in metaplasia, although one study refers to deacetylated histone H4 in areas of intestinal metaplasia of the stomach (Ono et al, 2002). HDAC inhibitors, together with DNA methyltransferase inhibition, enhance the reprogramming efficiency of human somatic cells into pluripotent stem cells (Dhanwani et al, 2011). Also, HDAC activity has been shown to be modulated by inflammatory stimuli (Villagra et al, 2010). It is thus likely that the inflammatory environment in which metaplasia arises also alters chromatin modification patterns, contributing to the process of metaplastic conversion.

## Concluding remarks

**Figure 3:**
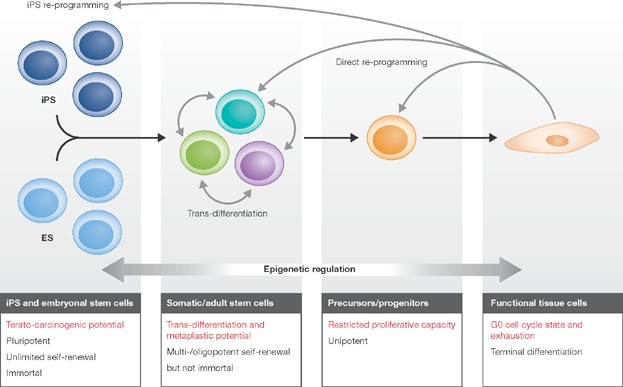
**Summary of cellular plasticity by reprogramming and trans-differentiation**Hierarchical and step-wise cell differentiation (black arrows; from left to right) can be reverted or rerouted by expression of cell type specifying transcription factors (grey arrows). The outcome of reprogramming or trans-differentiation depends on the transcription factors applied. Pluripotency of iPS cells, including terato-carcinogenic potential, can be achieved by expression of the four ‘Yamanaka factors’. ES or iPS can be re-differentiated into various cell types. Somatic or adult stem cells normally give rise to functional tissue cells through defined precursor/progenitor states with limited proliferative capacity. Somatic stem cells are quasi-stable entities residing in stem cell niches and maintain metaplastic trans-differentiation capacity. Somatic stem cells and specific cell types can be obtained by direct re-programming without passing through a complete de-differentiation, pluripotent state. Epigenetic changes are intrinsically linked with differentiation/de-differentiation programs and alteration of cell fate.

Transcription factors with nodal functions are involved in generation of somatic stem cells and steering precursor cells from an immature state into a fully functional mature and specialized state. Experimentally induced changes in the expression of transcription factors showed that it is possible to re-direct differentiation pathways in an artificial procedure coined reprogramming. Accumulating evidence suggests that reprogramming or trans-differentiation events are also ubiquitous in neoplastic conversion. The conceptual relationships between reprogramming and trans-differentiation cues are summarized in Fig 3.

A set of data shows that key transcription factors are often deregulated during leukaemogenesis and lymphomagenesis in conjunction with an ambiguous phenotype. In epithelia, cell fate is altered by inflammation-induced changes, involving key transcription factors that may be instrumental to formation of metaplastic foci that are since long thought of as sites of epithelial cancer initiation. A large body of circumstantial information suggests that such ambivalent phenotypes may be considered to play a crucial role in tumour initiation, although it remains to be resolved whether and how exactly a developing cancer cell profits from lineage promiscuity/ambiguity. A central question then is whether ‘reprogramming’ into a lineage promiscuous state only reflects the meltdown of epigenetic memory and/or confer selective advantages to tumour cells. In the case of biphenotypic leukaemia/lymphoma, lineage confused cells may combine the best out of two worlds, such as gain of growth factor autonomy by assimilating lineage divergent lymphoid/myeloid signalling pathways and growth factor sources, or/and by enhancing resistance to stress and apoptosis (Ding & Morrison, 2013; Greenbaum et al, 2013; Lamprecht et al, 2010; Tagoh et al, 2006). Unravelling the molecular genetic mechanisms involved in reprogramming and trans-differentiation will thus strongly advance our knowledge of cell differentiation, disclose mechanisms and novel targets in tumour medicine, and hopefully define the ingredients of reprogramming potions that prevent hacking cell differentiation programs, metamorphosing ‘Dr. Jekyll’ stem cells into dark side ‘Mr. Hyde’ transformants (R.L. Stevenson, 1886, ‘Strange case of Dr Jekyll and Mr Hyde’).

**Pending issues**

To what extent is trans-differentiation and lineage confusion a common feature of cancer onset and progression, of which metaplasia and leukaemic lineage ambiguities are the most observable reflexions?

Can we restrict tumour progression by targeting the lineage specific transcription factors responsible for this phenomenon?

Can therapeutic approaches take advantage by targeting multi-lineage features?

## Glossary

### Haematopoiesis

Haematopoiesis is the process of formation of several blood cell lineages through differentiation from haematopoietic stem cells. It occurs in adult humans mainly in the bone marrow, but can also occur in secondary lymphoid organs such as the thymus or spleen.

### Lineage confusion

Rrefers to a feature of some cancer cells displaying lineage infidelity/promiscuity/trans-differentiation that might confer a selective advantage to tumour growth.

### Lineage infidelity

McCulloch has introduced the term in 1983 (Smith et al, 1983) observing that acute leukaemia could entail markers of different haematopoietic lineages. Lineage infidelity correlated with failure of remission and was attributed to abnormal gene expression.

### Lineage promiscuity

Transient bi- or multiphenotypic stem cell stages that are preserved as a relic in leukaemic blast populations due to the primary oncogenic event that is involved in maturation arrest (Greaves et al, 1986).

### Metaplasia

A type of trans-differentiation, is the pre-neoplastic but often reversible physiological conversion of a given cell type into another in the context of tissue injury or chronic inflammation. Metaplasia gives rise to patches of a differentiated tissue amidst another tissue. Barrett's oesophagus, gastric intestinal metaplasia, squamous lung metaplasia or acinar-to-ductal metaplasia of the pancreas, are examples of metaplasia.

### Trans-differentiation/reprogramming/direct reprogramming

Although several definitions have been advanced, here trans-differentiation and reprogramming are mostly treated as analogous concepts of changing cell fate. All terms can be defined as the conversion of one differentiated cell type into another. While reprogramming solely is a concept of experimental manipulation that converts cells back to a quasi-embryonic like state, direct reprogramming brings forth a new differentiated phenotype, similar to trans-differentiation that may also occur under physiological or pathological conditions in the organism.
